# Understanding illness perception in pulmonary tuberculosis patients: One step towards patient-centered care

**DOI:** 10.1371/journal.pone.0218106

**Published:** 2019-06-12

**Authors:** Jinsoo Min, Chaeuk Chung, Sung Soo Jung, Hye Kyeong Park, Sung-Soon Lee, Ki Man Lee

**Affiliations:** 1 Division of Pulmonary and Critical Care Medicine, Department of Internal Medicine, Daejeon St. Mary’s Hospital, College of Medicine, The Catholic University of Korea, Seoul, Republic of Korea; 2 Division of Pulmonary and Critical Care Medicine, Department of Internal Medicine, Chungnam National University Hospital, Daejeon, Republic of Korea; 3 Division of Pulmonary and Critical Care Medicine, Department of Internal Medicine, Ilsan Paik Hospital, Inje University College of Medicine, Goyang, Republic of Korea; 4 Department of Internal Medicine, Chungbuk National University College of Medicine, Cheongju, Republic of Korea; 5 Division of Pulmonary & Critical Care Medicine, Department of Internal Medicine, Chungbuk National University Hospital, Cheongju, Republic of Korea; The University of Georgia, UNITED STATES

## Abstract

**Background:**

Assessing the illness perception of patients with tuberculosis (TB) could improve our understanding of their beliefs about disease and help address problems in their health-seeking behavior.

**Study aim:**

We assessed illness perception in patients with pulmonary TB in association with patients’ demographic, socioeconomic, and clinical features.

**Methods:**

Adult patients who were newly diagnosed with pulmonary TB at three tertiary hospitals in South Korea were included from November 2016 and September 2018. Participants’ illness perception was assessed using the Brief Illness Perception Questionnaire (BIPQ) at the start of anti-TB treatment.

**Results:**

In total, 390 patients with pulmonary TB completed this survey. The mean BIPQ score was 31.6 ± 13.2, and that was positively correlated with clinical TB scores. Patients were highly concerned about their illness, but believed in the treatment. Unhealthy eating habits were mentioned as the most prevalent perceived cause. Coughing for more than one month and alarming symptoms were significantly associated with BIPQ scores ≥ 33. Non-adherent patients had significantly higher BIPQ scores.

**Conclusions:**

Assessing the illness perceptions of those with severe TB-related symptoms and signs may help to identify TB patients with vulnerable to poor treatment outcomes.

## Introduction

Tuberculosis (TB) has existed for millennia and remains a major global health problem. It causes ill-health in approximately 10 million people each year and is one of the top ten causes of death worldwide[1]. After the development of anti-TB drugs in the early 20^th^ century that led to the development of the ‘prevention starts with cure’ paradigm, numerous public health interventions, including the DOT (Directed Observed Treatment Short-Course) strategy were introduced to improve treatment outcomes, thus reducing TB incidence. Although TB is now much better understood, fear and stigma persist among patients and their families[2]. Stigma is a social determinant of health, found to be a major barrier to accessing healthcare and the ability to manage illness and complete treatment[3].

Illness perception is a term used to refer to the mental presentations and personal ideas that people have about an illness. It is an important determinant of behavior and has been associated with numerous outcomes, such as treatment adherence and functional recovery[4]. Previous research showed that assessing the illness perception of patients with TB could address problems in patients’ health-seeking behavior that may disturb treatment adherence[5]. In addition, it would allow us a better understanding of patients’ beliefs about their disease and condition that might lead to stigmatization during the course of anti-TB treatment.

World Health Organization (WHO) acknowledged the importance of the ‘patient-centered approach’ in line with Pillar 1 of the END TB Strategy[6]. The first pillar focuses on providing universal access to TB care and prevention with greater attention to vulnerable and hard-to-reach populations. As a part of patient-centered care, the WHO recommends that educational, emotional, and economic supports should be provided to enable TB patients to complete the diagnostic process and full course of prescribed treatment. Accordingly, research evaluating illness perception in TB patients is essential to design and monitor any interventions that support care for patients on TB treatment. The Brief Illness Perception Questionnaire (BIPQ) was designed to provide simple and rapid assessment of illness perceptions and has been widely used in diverse ages, illness types, and languages[7].The objective of this study is to assess illness perception in patients with pulmonary TB by the implementation of the BIPQ at the start of an anti-TB treatment.

## Materials and methods

### Study design and subjects

A survey was prospectively conducted to assess the illness perception of patients with pulmonary TB in correlation with epidemiological and clinical data at the start of anti-TB treatment. This observation study was implemented as a part of a prospective cohort study in order to enroll patients with pulmonary TB at three university-affiliated tertiary hospitals in South Korea[8]. These hospitals participate in the public-private matrix (PPM) projects for TB control in South Korea. Trained nurses under the PPM project educate TB patients and monitor them for medication adherence and adverse drug reactions. Adult patients (age ≥ 19 years) who were newly diagnosed with pulmonary TB were included in this cohort from November 2016 to September 2018. Approximately 500 TB patients were diagnosed and treated annually in the participating hospitals. The sample size was calculated based on a population size of 1000 during the two-year study period with 5% margin of error at a 95% confidence level. The minimal required sample size was 278.

### Data collection

Demographic, socioeconomic, and clinical data were prospectively collected from enrolled patients using a case report form upon study entry. As a part of the assessment of clinical symptoms and signs, a clinical TB score[9] was also used at baseline. The TB score is a simple clinical score to measure the clinical status of TB patients during anti-TB treatment without using advanced technical equipment. TB score components included five self-reported symptoms (i.e. cough, dyspnea, night sweats, hemoptysis, and chest pain) and six clinical signs (anemic conjunctivae at eye-examination, positive finding in lung auscultation, a body temperature >37.0°C, pulse rate > 90 beats per minute, body mass index (BMI), and mid-upper arm circumference (MUAC)). The score was based on these 11 clinical variables, each contributing one or two points, with the maximum possible score being 13. If a patient does not complain about any symptom nor presents with positive findings in the physical examinations, the score of the corresponding variables will be zero. Patients with initial high TB scores are associated with a more severe clinical presentation.

Participants’ illness perception was assessed using the BIPQ[10] between first and second weeks of anti-TB treatment. The BIPQ is designed to provide a simple and rapid assessment of illness perception. It has eight items on a scale from 0 to 10 to assess the following dimensions; cognitive presentations such as consequences (item 1), timeline (item 2), personal control (item 3), treatment control (item 4), as well as identity (item 5), emotional representations such as concern (item 6), emotions (item 8), and illness comprehensibility (item 7). We used the original instructions to score the BIPQ. A higher score indicates a more threatening view of the illness. The scores of items 3, 4, and 7 are calculated and expressed as reverse scores in this scale. In order to assess causal representation, an open-ended response item (item 9) was used, which asked patients to list the three most important causal factors for their illness. All the responses to item 9 were further categorized according to the revised illness perception questionnaire, which listed 18 possible causes of illness, such as stress, germs, eating habits, pollution, aging, overwork, personality, smoking, etc.[11].The BIPQ was previously translated to and validated in Korean[12].

Medication adherence was also assessed by a simple questionnaire[13] at week 4. The medication adherence questionnaire contains four items: “Do you ever forget to take your medicine?”, “Are you careless at times about taking your medicine?”, “When you feel better, do you sometimes stop taking your medicine?”, and “Sometimes if you feel worse when you take the medicine, do you stop taking it?”. Response categories are ‘yes’ or ‘no’. A person was defined to be non-adherent if he or she responded affirmatively to at least one question.

### Statistical analysis

Continuous variables are presented as means and standard deviations or medians and interquartile ranges (IQR), whereas the discrete variables are presented as frequencies or percentages. Pearson’s correlation coefficient was calculated to determine correlations between each item of the BIPQ and TB score. After adding scores of all the BIPQ items, the correlation between the total BIPQ score and TB score was also tested. We transformed the total BIPQ score into a dichotomous variable based on the median value of the total BIPQ score and compared the characteristics between people with high and low total BIPQ scores. In order to identify variables associated with pre-defined high total BIPQ scores, univariate analysis was performed using the chi-square test, and multivariate analysis was performed using a binary logistic regression. In the regression analysis, forward and backward methods were used to select the variables included in the final model. Finally, mean values of the BIPQ items between adherent and non-adherent groups were compared using the independent-samples t-test. All statistical analyses were performed using SPSS version 15.0 (SPSS, Chicago, IL, USA) and P < 0.05 was considered statistically significant.

### Ethical statement

This study was performed in accordance with the Declaration of Helsinki. The protocol and informed consent forms are approved for their scientific content and compliance with human subject research regulations by the Institutional Review Boards of Chungbuk National University Hospital. All adult participants provided written informed consent to participate in this study.

## Results

**Fig 1:**
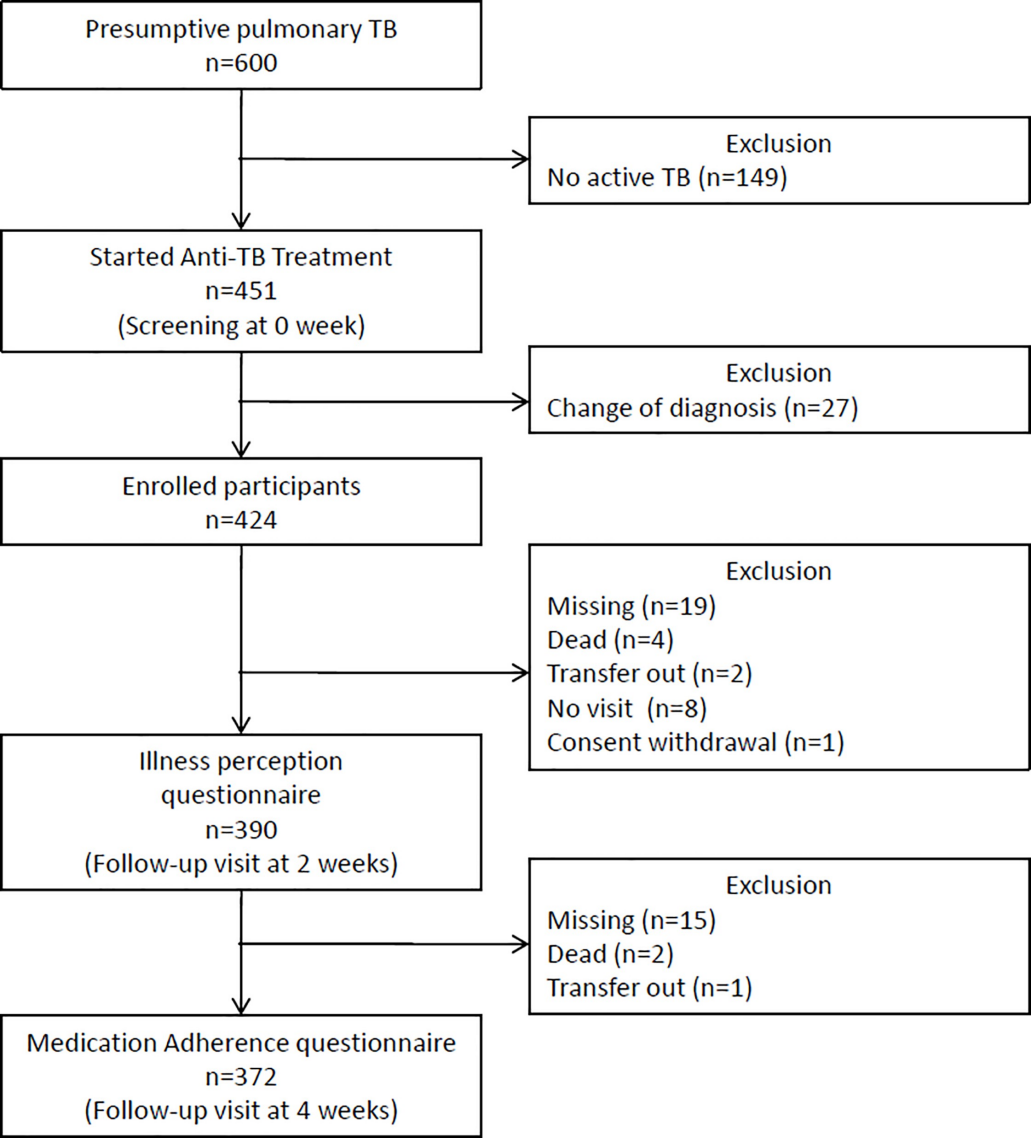
Enrollment flow-chart.

After screening 600 patients with presumptive pulmonary TB, 424 patients were diagnosed with pulmonary TB and enrolled in this study (Fig 1). The completion rate for the BIPQ was 92.0% (390/424). Table 1 summarizes the demographic, socioeconomic, and clinical characteristics of 390 study participants. One fifth of the patients had a previous history of anti-TB treatment. The number of patients with culture-positive pulmonary TB was 268 (68.7%). The mean patient age was 58.5 ± 19.5, with 242 males (62.1%) and 156 females (37.9%). The mean clinical TB score was 2.5 ± 1.8 at baseline, and the components of TB score and their results are shown in Table 2.

**Table 1:** Baseline characteristics of 390 patients with pulmonary tuberculosis.

Characteristics	All patients (n = 390)
Male	242 (62.1%)
Age, years	58.5 ± 19.5
Body Mass Index, kg/m^2^	21.4 ± 3.0
Current smoker	104 (26.7%)
Drinking history within 1 year	181 (46.4%)
Education	
Low (primary or middle school)	170 (43.6%)
High (middle school or university)	220 (56.4%)
Income	
Low (<1,500,000KRW)	261 (66.9%)
High (≥1,500,000KRW)	129 (33.1%)
Job	
Service industry	99 (25.4%)
Labor works	70 (17.9%)
Unemployed	221 (56.7%)
Previous tuberculosis history	80 (20.5%)
Presence of comorbidities	229 (58.7%)
Symptoms	
Cough for > 1 month	121 (31.0%)
Alarming symptoms (hemoptysis, dyspnea, or chest pain)	141 (36.2)
Microbiologic tests	
Positive AFB smear test result	107 (27.4%)
Positive AFB culture test result	268 (68.7%)
Bilateral disease on Chest radiograph	100 (25.6%)

Data are presented as mean ± standard deviation or no. (%).

KRW, Korean Won; AFB, acid-fast bacilli

**Table 2:** Clinical characteristics and TB score of 390 patients with pulmonary tuberculosis.

Parameters	
Cough	239 (61.3%)
Hemoptysis	36 (9.2%)
Dyspnea	86 (22.1%)
Chest pain	51 (13.1%)
Night sweating	46 (11.8%)
Anemic conjunctivae	10 (2.6%)
Heart rate > 90beats per minute	118 (30.3%)
Positive finding at lung auscultation	157 (40.3%)
Body temperature>37.0°C	116 (29.7%)
Body mass index (kg/m^2^)	
< 18.0	37 (9.5%)
< 16.0	8 (2.1%)
Middle upper arm circumference (mm)	
< 220	37 (9.5%)
< 200	15 (3.8%)
TBscore	
0~2	210 (53.9%)
3~4	128 (32.8%)
5~	52 (13.3%)
Mean ± standard deviation	2.5 ± 1.8
Median [interquartile range]	2 [1–3]

Data are presented as mean ± standard deviation, median with interquartile range or no. (%).

The mean and median values of the BIPQ score were 31.6 ± 13.2 and 33.0 (IQR, 23.0–41.3), respectively. We found positive correlations between the BIPQ score and TB score (p < 0.001). Mean values for the BIPQ items and their correlations with the TB score are presented in Table 3. Among eight items of the BIPQ, ‘concern’ (item 6) had the highest score, that indicated that the participants were highly concerned about their illness, followed by ‘consequence’ (item 1), indicating that the participants worried that illness would have an adverse effect on their life. ‘Timeline (duration of illness)’ and ‘identity (experience of symptoms correctly referred to TB)’ also scored high, which meant that patients believed that TB would last long and experienced many symptoms associated with TB. ‘Treatment control’ (item 4) had the lowest BIPQ score that meant that participants regarded anti-TB treatment as being helpful in the management of their illness.

**Table 3:** Mean and median values of brief illness perception questionnaire items and correlation with clinical TB score at baseline.

Items	Mean ± S.D	Median [IQR]	Correlation^a^	p-value
1. Consequence	5.1 ± 3.3	5 [2–8]	0.191	0.000
2. Timeline	4.5 ± 2.7	5 [2–6]	0.222	0.000
3. Personal control§	2.6 ± 2.6	2 [0–5]	0.037	0.466
4. Treatment control§	2.4 ± 2.4	2 [0–4]	-0.036	0.476
5. Identity	3.3 ± 3.0	3 [1–5]	0.276	0.000
6. Concern	5.4 ± 3.4	5 [2–9]	0.155	0.002
7. Coherence§	3.9 ± 2.8	4 [2–5]	0.004	0.940
8. Emotional representation	4.3 ± 3.2	4 [1.75–7]	0.093	0.068
Total score of BIPQ	31.6 ± 13.2	33.0 [23.0–41.3]	0.219	0.000

§ The scores of item 3,4, and 7 are expressed as reverse scores.

S.D, standard deviation; IQR, interquartile range; BIPQ, brief illness perception questionnaire

^a^ Pearson’s correlation coefficient

For the open-ended questions regarding patients’ opinion on the possible causes of their illness, 238 (61.0%) participants responded with 439 answers, 88 (22.6%) participants stated that they did not know the causes, and 64 (16.4%) participants did not respond. ‘Unhealthy diet or eating habit’ was the most frequent response, followed by ‘altered immunity’, ‘germ or virus’, ‘stress or worry’, and ‘smoking’ (Table 4).

**Table 4:** Responses of possible causes of participant’s illness.

Possible causes	n (%)
Unhealthy diet or eating habit	85 (19.4%)
Altered immunity	51 (11.6%)
Stress or worry	47 (10.7%)
Germ or virus	46 (10.5%)
Smoking	34 (7.7%)
My own behavior	30 (6.8%)
Pollution in the environment	30 (6.8%)
Overwork	28 (6.4%)
Fatigue	26 (5.9%)
Alcohol	25 (5.7%)
Poor medical care in my past	16 (3.6%)
Hereditary	8 (1.8%)
My emotional state / personality	7 (1.6%)
Chance or bad luck	5 (1.1%)
Accident or injury	1 (0.2%)

Female participants had significantly higher BIPQ scores than male participants (33.7 ± 11.8 vs. 30.4 ± 13.9, p-value = 0.014) (Table 5). Coughing for more than a month, alarming symptoms (i.e. hemoptysis, dyspnea, or chest pain), and high TB scores were associated with the BIPQ score ≥ 33 upon univariate analysis. Multivariate analysis showed that the job related service industry (adjusted odds ratio [OR] = 2.26, 95% confidence interval [CI] = 1.27–4.01), coughing for more than one month (adjusted OR = 1.58, 95% CI = 1.00–2.50), and alarming symptoms (adjusted OR = 1.87, 95% CI = 1.21–1.89) were significantly associated with the BIPQ score ≥ 33 (Table 6). Using the same multivariate analysis mode replacing coughing for more than one month and alarming symptoms with the TB score, it was found that participants with a TB score ≥ 5 were three times more likely to have a BIPQ score of≥ 33 (adjusted OR = 3.07, 95% CI = 1.6–6.0).

**Table 5:** Brief illness perception questionnaire scores of patients with pulmonary tuberculosis according to their baseline characteristics.

Characteristics		All patients (n = 390)	BIPQ score	p-value	BIPQ score		p-value^a^
					≥ 33 (n = 196)	< 33 (n = 194)	
Gender	Male	242 (62.1%)	30.4 ± 13.9	0.014	116 (59.2%)	126 (64.9%)	0.241
	Female	148 (37.9%)	33.7 ± 11.8		80 (40.8%)	68 (35.1%)	
Age, years	≥65	161 (41.3%)	31.2 ± 13.9	0.594	80 (40.8%)	81 (41.8%)	0.851
	<65	229 (58.7%)	31.9 ± 12.8		116 (59.2%)	113 (58.2%)	
Body mass index, kg/m^2^	<18.5	59 (15.1%)	33.5 ± 12.4	0.253	32 (16.3%)	27 (13.9%)	0.507
	≥18.5	331 (84.9%)	31.3 ± 13.4		164 (83.7%)	167 (86.1%)	
Current smoker	Yes	104 (26.7%)	32.3 ± 14.1	0.926	53 (27.0%)	51 (26.3%)	0.867
	No	286 (73.3%)	31.4 ± 13.0		143 (73.0%)	143 (73.7%)	
Drinking history within 1 year	Yes	181 (46.4%)	31.0 ± 13.1	0.356	89 (45.4%)	92 (47.4%)	0.690
	No	209 (53.6%)	32.2 ± 13.3		107 (54.6%)	102 (52.6%)	
Marriage	Yes	319 (81.9%)	31.2 ± 13.5	0.136	155 (79.1%)	164 (84.5%)	0.163
	No	71 (18.2%)	33.8 ± 12.0		41 (20.9%)	30 (15.5%)	
Education	Low	170 (43.6%)	31.5 ± 13.6	0.850	83 (42.3%)	87 (44.8%)	0.619
	High	220 (56.4%)	31.8 ± 13.0		113 (57.7%)	107 (55.2%)	
Income	Low	261 (66.9%)	32.2 ± 13.0	0.207	129 (65.8%)	132 (68.0%)	0.641
	High	129 (33.1%)	30.4 ± 13.6		67 (34.2%)	62 (32.0%)	
Job	Service industry	99 (25.4%)	33.5 ± 13.3	0.272	58 (29.6%)	41 (21.1%)	0.118
	Labor works	70 (17.9%)	30.6 ± 14.6		36 (18.4%)	34 (17.5%)	
	Unemployed	221 (56.7%)	31.2 ± 12.7		102 (52.0%)	119 (61.3%)	
Previous TB history	Yes	80 (20.5%)	31.9 ± 13.7	0.843	39 (19.9%)	41 (21.1%)	0.762
	No	310 (79.5%)	31.6 ± 13.1		157 (80.1%)	153 (78.9%)	
Comorbidity	Yes	229 (58.7%)	31.9 ± 14.1	0.632	115 (58.7%)	114 (58.8%)	0.986
	No	161 (41.3%)	31.3 ± 12.0		81 (41.3%)	80 (41.2%)	
AFB smear test result	Positive	107 (27.4%)	33.0 ± 12.6	0.222	62 (31.6%)	45 (23.2%)	0.062
	Negative	283 (72.6%)	31.1 ± 13.5		134 (68.4%)	149 (76.8%)	
AFB culture test result	Positive	268 (68.7%)	32.0 ± 13.3	0.459	143 (73.0%)	125 (64.4%)	0.069
	Negative	122 (31.3%)	30.9 ± 13.1		53 (27.0%)	69 (35.6%)	
Bilateral disease on chest radiograph	Yes	100 (25.6%)	30.3 ± 13.3	0.231	46 (23.5%)	54 (27.8%)	0.324
	No	290 (74.4%)	32.1 ± 13.2		150 (76.5%)	140 (72.2%)	
Coughing for 1 month	Yes	121 (31.0%)	34.5 ± 12.8	0.005	72 (36.7%)	49 (25.3%)	0.014
	No	269 (69.0%)	30.4 ± 13.3		124 (63.3%)	145 (74.7%)	
Alarming symptoms	Yes	141 (36.2%)	34.6 ± 13.2	0.001	84 (42.9%)	57 (29.4%)	0.006
	No	249 (63.8%)	29.9 ± 13.0		112 (57.1%)	137 (70.6%)	
Heart rate > 90beats per minute	Yes	118 (30.3%)	33.8 ± 12.4	0.031	65 (33.2%)	53 (27.3%)	0.209
	No	272 (69.7%)	30.7 ± 13.5		131 (66.8%)	141 (72.7%)	
Body temperature>37.0°C	Yes	116 (29.7%)	34.0 ± 12.8	0.023	66 (33.7%)	50 (25.8%)	0.088
	No	274 (70.3%)	30.6 ± 13.3		130 (66.3%)	144 (74.2%)	
Clinical TB score	0~2	208 (53.3%)	29.2 ± 13.3	0.003	37 (18.9%)	16 (8.2%)	0.003
	3~4	129 (33.1%)	33.1 ± 13.6		68 (34.7%)	61 (31.4%)	
	5~	53 (13.6%)	37.5 ± 9.7		91 (46.4%)	117 (60.3%)	

TB, tuberculosis; AFB, acid-fast bacillus; BIPQ, brief illness perception questionnaire

^a^ chi-square test

**Table 6:** Multivariate analysis assessing risk factors for brief illness perception questionnaire score ≥ 33.

		BIPQ ≥ 33 (n = 196)	BIPQ < 33 (n = 194)	Adjusted OR^a^ (95% CI)	p-value
Gender	Female	80 (40.8%)	68 (35.1%)		
	Male	116 (59.2%)	126 (64.9%)	0.73 (0.48–1.12)	0.154
Age, years	<65	116 (59.2%)	113 (58.2%)		
	≥65	80 (40.8%)	81 (41.8%)	1.24 (0.76–2.03)	0.381
Job categories	Unemployed	102 (52.0%)	119 (61.3%)		
	Labor works	36 (18.4%)	34 (17.5%)	1.48 (0.82–2.68)	0.193
	Service industry	58 (29.6%)	41 (21.1%)	2.26 (1.27–4.01)	0.006
AFB smear test results	Negative	134 (68.4%)	149 (76.8%)		
	Positive	62 (31.6%)	45 (23.2%)	1.54 (0.96–2.48)	0.071
Coughing for >1month	No	124 (63.3%)	145 (74.7%)		
	Yes	72 (36.7%)	49 (25.3%)	1.58 (1.00–2.50)	0.048
Alarming symptoms	No	112 (57.1%)	137 (70.6%)		
	Yes	84 (42.9%)	57 (29.4%)	1.87 (1.21–2.89)	0.005

BIPQ, brief illness perception questionnaire; OR, odds ratio; CI, confidence interval; AFB, acid-fast bacilli

^a^ multiple logistic regression

Among 372 participants who completed the medication adherence questionnaire (Table 7), 59 (15.9%) were identified as non-adherent. Those identified as non-adherent had significantly higher BIPQ scores (34.9 ± 9.8 vs. 31.2 ± 13.8; p-value = 0.014). Of the eight items of the BIPQ, ‘consequence’ (item 1) and ‘timeline’ (item 2) had the highest BIPQ scores in the non-adherent group.

**Table 7:** Comparison of brief illness perception questionnaire score between non-adherent and adherent patients.

	Non-adherence (n = 59)	Adherence (n = 313)	p-value^a^
Consequence	6.1 ± 2.9	4.9 ± 3.4	0.006
Timeline	5.3 ± 2.3	4.4 ± 2.7	0.016
Personal control	2.8 ± 2.7	2.6 ± 2.6	0.643
Treatment control	2.6 ± 2.4	2.3 ± 2.3	0.412
Identity	3.4 ± 3.0	3.4 ± 2.9	0.856
Concern	5.8 ± 3.4	5.4 ± 3.4	0.382
Coherence	4.1 ± 2.5	3.8 ± 2.8	0.449
Emotional representation	5.0 ± 2.7	4.3 ± 3.3	0.075
Total score of BIPQ	35.2 ± 9.9	31.2 ± 13.8	0.008

^a^Independent-sample t-test

## Discussion

To the best of our knowledge, this is the first prospective multicenter study to assess illness perception and its association with clinical presentation and adherence, based on a large sample of TB patients. Illness perceptions are the organized cognitive representations or beliefs that patients have about their illness, and it is an important determinant of health-seeking behavior[4]. However, patient’s beliefs are often different from those treating patients. Most healthcare professionals often neglect patients’ ideas and thoughts concerning their illness, and do not understand the causes of patient disbelief and non-compliance to TB care. In addition, illness perception varies depending on demographic factors, socioeconomic status, and geographic and cultural variations. In our study, the BIPQ score at baseline was concordant with the clinical TB score at the time of TB diagnosis. Patients who were coughing for more than one month and presenting with alarming symptoms had more threatening view of their illness. Among the BIPQ items, consequence, timeline, identity, and concern were significantly positively correlated with TB scores. Based on our results, the assessment of illness perception for those initially presenting with severe symptoms or signs would help to address problems in patient behavior that may disturb successful treatment outcomes. However, since the correlation between the BIPQ and clinical TB score was relatively modest, which is similar to another multicenter study [5], we have to be cautious not to miss TB patients with minimal respiratory symptoms and signs who are at risk of poor health-seeking behaviors.

TB has been traditionally regarded as a disease of the poor[14]. Many characteristics of low socioeconomic status, for example overcrowding and malnutrition, are established individual-level risk factors[15]. TB as a social disease further is associated with stigma, discouraging patients to seek out healthcare systems. TB is labeled as a marker for human immunodeficiency virus in high prevalence settings, leading to distinct forms of double stigma[3]. In our study, ‘concern’ was ranked the highest, followed by ‘consequence’, ‘timeline’, and ‘emotional presentations’. It conveys that patients were very concerned about their illness and that their illness would continue for a long time and, in turn, has adversely affected their life and emotions. Despite the patients’ high trust in their treatment, expressed by their lowest score on ‘treatment control’ in our study, many patients were worried about TB, which might be caused by the historical community norm that perceived TB as the incurable. These low-perceived items might render TB patients more vulnerable to stigma, and as TB stigma would result in a sense of shame or guilt, it could lead to self-isolation as TB-infected individuals internalize their community’s negative judgments about the disease[16]. The TB community has always recognized the stigma that surrounds the disease, but has done far less to confront it[17]. TB stigma is still regarded as either natural or intractable, or easily overcome with a general improvement in quality or access to care[18]. The assessment of illness perceptions using a simple questionnaire might help us detect TB patients vulnerable to stigma in advance and provide evidence to plan operational research and implement suitable interventions.

In the review of literature, there were several studies evaluating illness perceptions of TB. The BIPQ scores of a multicenter study[5] from Europe and Asia between 2008 and 2011 and a single-center study[19] from Sri Lanka between 2014 and 2015were 36.3 ± 11.1 and 27.4 ± 14.4, respectively, indicating that illness perception varies depending on socio-demographic and geographic backgrounds. However, these two studies revealed that concern had the most threatening view among the BIPQ items, similar to our study. Another single-center study[20] from South Korea in 2009 showed that the BIPQ score was 48.9±14.8, and that is much higher than our results. This decrease in the BIPQ score from 2009 to 2018 in South Korea might be attributable to the successful implementation of a national TB control program in South Korea[21]. Since 2011, Korea has strengthened its TB policy with strong political will and has shown a 5.2% reduction annually in the incidence of newly reported TB cases from 2011 to 2016. If we can evaluate these temporal changes of BIPQ scores over an extended period, we can acquire a more insightful perspective on illness perceptions of TB.

Many researchers have identified several factors determining adherence to treatment, such as poverty and the financial burden of treatment; gender; working conditions and migration for work; education level and knowledge, attitudes and beliefs about treatment; degree of family and community support; and organization of services[15]. Treatment adherence is a complex behavioral issue and a full understanding of the factors that prevent patients taking anti-TB drugs correctly and regularly is essential for improving anti-TB treatment outcomes. Based on our study results, non-adherent patients had higher BIPQ scores than adherent patients. In addition, non-adherent patients had more threatening views on two items of the BIPQ, ‘consequence’ and ‘timeline’. A previous systemic review[22] found that knowledge, attitudes and beliefs about treatment and care can act as a filter for the information and treatment offered in the healthcare services. The influence of patients’ interpretation of the disease and illness on their adherence behavior has been documented previously, and understanding illness perception of patients, that are often different from that of healthcare providers, will help identify those patients with risks of non-adherence.

For the open-ended questions on the causal item, unhealthy behaviors, such as unhealthy eating habit, lack of exercise, overwork, smoking, and alcohol, were the most mentioned causes in our study. Physical, psychological, and emotional exhaustion, such as stress, worry, fatigue, altered immunity, and personality, were also important categories. TB has been a mysterious disease for a long time before the discovery of the TB bacillus in the late 19^th^ century, generating false knowledge and inappropriate beliefs concerning TB. Indeed, almost two fifth of participants could not specify the main cause of pulmonary TB in our study. A small number of participants denied having TB and ascribed it to bad luck or chance. Educating patients and raising awareness in the community are key components of the END TB Strategy by the WHO; however, such patient-centered support programs are limited, indicating that further research will be needed to identify and verify effective and efficient support interventions for TB patients[23].

Based on the present findings, we have listed several future research areas: (1) identifying high-risk patients vulnerable to TB stigma in various cultural settings, (2) evaluating knowledge about and attitude towards TB of patients with different backgrounds, and (3) assessing the relationship between underprivileged patients and poor treatment outcome. These future research topics are warranted in order to understand and effectively manage illness perception of vulnerable TB patients. Process evaluation and sophisticated qualitative methods will be required to plan multidisciplinary social support intervention during a course of anti-TB treatment[3]. For example, health-promotion campaigns using user-friendly modalities such as smartphones will be helpful to reinforce the belief that the disease is widespread and treatable to reduce stigma and improve health-seeking behavior [2].

The major strength of the present study is its relatively large sample size with a high response rate. Data were collected prospectively by trained TB nurses. The survey was conducted using a previously validated and recognized tool. After translation and validation of the Korean version[12], we set additional research objectives and planned to conduct this current study in order to evaluate risk factors for high BIPQ scores and assess the relationship between BIPQ and medication adherence.

However, the present study has a couple of limitations that should be considered. First, the survey was conducted in university-affiliated tertiary hospitals that actively participate in the national PPM project. In 2016, 66% of newly notified TB patients were treated and managed at the PPM collaboration hospitals[21]. Our results thus arguably cannot be inferred to other TB clinics, such as public health centers and non-PPM private hospitals, in South Korea. Secondly, we did not assess other variables that were known to be associated with illness perception. For example, depression and anxiety, which are common in TB patients, influence illness perception[19,24]. Another study identified positive correlations between quality of life and illness perception among TB patients[25]. Additionally, a well-designed qualitative research with the primary objective of evaluating its association with other social determinants of health and treatment outcomes will enhance our understanding of illness perception among TB patients. Thirdly, we did not compare illness perception to other acute respiratory infection, which was beyond the scope of our study objectives. Additional enrollment of patients with similar respiratory symptoms, but not diagnosed with TB, would broaden our understanding of patient’s perception toward TB.

In conclusion, we used a simple short questionnaire to assess illness perception and obtained initial information without difficulties. Based on our results, assessing illness perception in those with severe TB-related symptoms and signs at diagnosis might be beneficial to identify possible TB patients vulnerable to poor treatment outcomes. The concept of patient-centered care has received increased attention during the last decades by the TB community[26]. Understanding patients’ illness perception will provide sound evidence for planning patient-centered care and implementing supportive interventions.
